# Folate Deficiency during Early-Mid Pregnancy Affects the Skeletal Muscle Transcriptome of Piglets from a Reciprocal Cross

**DOI:** 10.1371/journal.pone.0082616

**Published:** 2013-12-09

**Authors:** Yi Li, Xu Zhang, Yanxiao Sun, Qiang Feng, Guanglei Li, Meng Wang, Xinxing Cui, Li Kang, Yunliang Jiang

**Affiliations:** 1 Laboratory of Animal Molecular Genetics, College of Animal Science, Shandong Agricultural University, Taian, China; 2 The Central Hospital of Taian, Taian, China; 3 Taian Hospital Affiliated to Taishan Medical University, Taian, China; 4 Laiwu Bureau of Animal Husbandry and Veterinary Medicine, Laiwu, China; Harbin Institute of Technology, China

## Abstract

Folate deficiency (FD) during pregnancy can cause fetal intrauterine growth restriction in pigs, of which the skeletal dysplasia is a major manifestation. Factors influencing muscle development are very important in the formation of porcine meat quality trait. However, the effect of folate deficiency on skeletal muscle development and its molecular mechanisms are unknown. The objective of this study is to determine the effect of maternal folate deficiency on the skeletal muscle transcriptome of piglets from a reciprocal cross, in which full-sibling Landrace (LR) and full-sibling Chinese local breed Laiwu (LW) pigs were used for reciprocal cross matings, and sows were fed either a folate deficient or a normal diet during early-mid gestation. In addition, the difference in the responsiveness of the piglets to folate deficiency during early-mid pregnancy between reciprocal cross groups was investigated. Longissimus dorsi (LD) muscle samples were collected from newborn piglets and a 4 × 44K Agilent porcine oligo microarray was used for transcriptome analysis of porcine LD muscle. The results showed that folate deficiency during early-mid pregnancy affected piglet body weight, LD muscle fiber number and content of intramuscular triglyceride. The microarray results indicated that 3154 genes were differentially expressed between folate deficient and normal piglets from the LR♂ × LW♀ cross, and 3885 differentially expressed genes (DEGs) in the ones from the LW♂ × LR♀ cross. From functional analyses, sow folate deficiency affected almost all biological processes in the progeny. Lipid metabolism-related genes and associated metabolic pathways were regulated extensively by folate deficiency, especially in LR♂ × LW♀ cross piglets. Most of the genes that are regulated by folate deficiency in the LD muscle of piglets were different between LR♂ × LW♀ and LW♂ × LR♀ crosses, suggesting some epigenetic effects of FD exist in genes underlying myogenesis and intramuscular fat deposition in piglets.

## Introduction

Improving meat quality has become a research focus for pig breeding experts in the 21^st^ century [1]. Meat quality is related to the expression of genes controlling myogenesis and lipid metabolism, which is affected by living conditions, nutrition, and other factors [2,3]. Recent evidence indicates that poor nutrition around the time of conception and during gestation can result in long-term modulation of gene expression and the physiological signaling pathways of the offspring [4-6]. Several studies have shown that maternal nutrient restriction during fetal development can affect the development of skeletal muscle and meat quality [7-9]. 

Folate (also called folic acid) is a water-soluble B vitamin (B9), and is an essential nutrient required for the *de novo* synthesis of deoxythymidine monophosphate (dTMP) and S-adenosyl methionine (AdoMet) through one-carbon metabolism and the methionine cycle, respectively. Many studies have demonstrated a link between folate deficiency during early pregnancy and developmental abnormalities, such as neural tube defects [10], abnormal embryonic development [11,12], and tumor formation [13]. For these reasons, dietary folate supplementation is routinely recommended during early pregnancy in human [14]. Another indication that folate status during early pregnancy can significantly change offspring phenotype is that folate supplementation during pregnancy in A^vy^ mice alters the fur color of their offspring [15]. In sheep, Sinclair et al. also reported that folate deficiency in pregnant ewes affected the development and deposition of their offspring’s fat by changing the DNA methylation patterns [16]. Therefore, inadequate folate status during pregnancy can cause DNA synthesis and methyl metabolism disorders, thus interfering with normal development or causing disease. 

Muscle fiber characteristics and intramuscular fat (IMF) content are the two most important factors influencing meat quality. Early pregnancy nutrition can affect fetal myogenesis and fat deposition, and these influences continue to adulthood [17-19]. Studies have shown that folate deficiency during pregnancy can cause fetal intrauterine growth restriction (IUGR) [20], while the skeletal dysplasia is a major manifestation of IUGR [21]. Nutritional analysis has shown that there is insufficient folate in the traditional diets of pregnant sows [22,23], and folate supplementation during pregnancy reduces embryonic mortality and markedly improves reproductive performance [24,25]. However, the effect of folate on skeletal muscle development and meat quality in pigs remains unknown, the skeletal muscle transcriptome and underlying molecular basis influenced by folate deficiency during pig pregnancy are not reported.

Microarrays have been used to investigate differential gene expression in pigs of different breeds and developmental stages at the transcriptome level, and have proved to be a powerful and direct tool for the study of known transcripts in complex developmental programs [26-28]. In this study, using the 4 × 44K Agilent porcine oligo microarray, we investigated the effects of folate deficiency during early-mid pregnancy on the skeletal muscle transcriptome of piglets from a reciprocal cross. Our study provides new insights into sow folate nutrition and muscle development of the offspring, and suggests a potential strategy to improve meat quality. In addition, our study provides some clues to the relationship between folate deficiency during pregnancy and the fetal muscular tissue development in human. 

## Materials and Methods

### Ethics Statement

All animal experiments were approved by the Institutional Animal Care and Use Ethics Committee of Shandong Agricultural University (Permit Number: NO. 2007005, Figure S1) and performed in accordance with the “Guidelines for Experimental Animals” of the Ministry of Science and Technology (Beijing, China). All surgery was performed according to recommendations proposed by the European Commission (1997), and all efforts were made to minimize suffering.

### Experimental design

A reciprocal cross between Landrace (LR) and Laiwu pigs (LW), i.e. LR♂ × LW♀ and LW ♂ × LR♀, was performed. LW pigs are a Chinese local pig breed that is characterized by a high intramuscular fat content. Four full-sibling LR pigs (three sows and one sire) and four full-sibling LW pigs (three sows and one sire) were used for the reciprocal cross. To examine the impact of folate deficiency during early-mid pregnancy on the muscle development of the progeny, the sows were divided into a folate deficient (FD) group and a normal folate (N) group. For each cross, two pregnant sows were treated with FD diet and one with N diet. In the FD group, standard pig feed without additional folate was provided to the pregnant sows from pregnancy. Premixes without multivitamin were used and multivitamin was mix respectively (Table 1). After 60 days of gestation, the sows were fed an ordinary feed designed for late pregnant sows. In the N group, the sows were fed standard pregnant sow feed (NRC 1998) with 1.3 mg/kg of added folate; this meets the folate requirement of pregnant sows. The composition of the diets was shown in Table 1. The sows had free access to water through the test. 

**Table 1:** Composition of the experimental diets.

Ingredients	Normal diet	Folate deficient diet
corn (kg)	54	54
Soybean meal (kg)	12	12
wheat bran (kg)	29.9	29.9
Vitamin E (g)	0.2	0.2
baking soda (kg)	0.1	0.1
premixes without multivitamin (kg)	3.97	3.97
Vitamin A (IU)	1350000	1350000
Vitamin D3 (IU)	225000	225000
Vitamin E (IU)	1556	1556
Vitamin K3 (MSB) (g)	0.3	0.3
Vitamin B1 (g)	0.18	0.18
Vitamin B2 (g)	0.6	0.6
Vitamin B6 (g)	0.03	0.03
Vitamin B12 (mg)	2.4	2.4
Biotin (mg)	9	9
Niacin / Niacinamide (g)	2.4	2.4
Calcium pantothenate (g)	1.5	1.5
folate (mg)**^*a*^**	130	0
feed weight (kg)	100	100

^a^ 1.3 mg/kg is the standard dietary folate requirement of pregnant sows.

After birth, the body length, body height and body weight of piglets were recorded. The longissimus dorsi (LD) samples were collected from the newborn piglets immediately, and washed briefly with PBS before being frozen in liquid nitrogen for detection. Three male piglets from each FD group and from each N group (altogether 12 individuals) were randomly selected for transcriptome analysis of skeletal muscle tissues using the 4 × 44K Agilent porcine oligo microarray. 

### Histochemical examination

Part of the LD muscle tissues was cut into 10 μm frozen section by HM550 freezing microtome, and then stained with haematoxylin and eosin (HE) for light microscopy. The muscle ﬁbre numbers in FD group and N group were calculated in ten ﬁelds (200×), and averaged data were used for calculations. While the diameter of 10 muscle ﬁbers was measured per ﬁeld (200×), and ten random ﬁelds were selected for quantiﬁcation. Similarly averaged data were used for calculations.

### Intramuscular triglyceride

According to the extraction method of Schenk [29] and Zhu [7], 0.5g of porcine LD muscle samples were homogenized in 10ml 2:1 chloroform/methanol after removing all visible fats. Triglycerides were extracted and saponiﬁed in 4% ethanolic KOH. Free glycerol in these samples was determined spectrophotometrically [7,29]. Pure glycerol was used as the standard for quantiﬁcation. The content of intramuscular triglyceride was expressed as millimoles of glycerol per kilogram of LD muscle.

### Total RNA preparation and microarray hybridization

Total RNA was isolated from frozen LD samples using TRIzol reagent (Invitrogen, USA) according to the manufacturer’s instructions and dissolved in RNase-free water. Total RNA concentration was assessed by spectrophotometry (OD 260 nm) and adjusted to a final concentration of 2.0μg/μl. The integrity and purity of the RNA were determined by the absorbance ratio at 260/280 nm and 2100 RIN. Microarray hybridization was carried out according to the instructions in the Agilent Expression Analysis Technical Manual. Comparison of the muscle transcriptomes of the FD and N groups was achieved using a 4 × 44K Agilent porcine oligo microarray that contains 42,034 probes for genes and transcripts publicly available through well-known databases such as Refseq, Unigene and TIGR (Shanghai Biotechnology Co., Ltd. China). The WEB-based Gene Set Analysis Toolkit was used for the categorization of Gene Ontology (GO) terms for biological processes. The arrays were scanned using the Scanner 3000 and the standard protocol was used for data extraction.

### Microarray data analyses and statistics

Raw spot intensities were first submitted to quality filtration based on four criteria: intensity, uniformity, saturation and outlier detection. The raw data from each transcript were subjected to log_2_ transformation. The distribution of the expressed genes was analyzed by JMP4.0 according to their expression level and gene expression flags were assigned. If a gene was flagged as “A” by the scanner based on the data normalization and results of the Agilent Microarray Suite 4.0 software, it was considered to be "not detected", and hence "not expressed" in this study. Similarly, the genes with “P” flags were considered to be “expressed transcripts”. Expressed transcripts were defined as being present in at least one sample and were used for all following studies. The expression value of each probe set was normalized and calibrated using the robust multi-array average (RMA) method. The microarray dataset have been submitted to ArrayExpress, and the accession number is E-MEXP-3996.

Analysis of variance (ANOVA) was used to identify genes with significant differences in expression between the FD and N groups. Screening of differentially expressed genes (DEGs) was performed on the basis of differences expressed during muscle development or lipid metabolism. Expression levels in the N groups were used as the control values, and comparisons were made with expression levels in the FD groups in the reciprocal cross. Genes were considered to be DEGs only when the fold-change (FC) in abundance for all comparisons exceeded 2.0 during screening.

### Functional analysis

Gene Ontology enrichment analysis was performed for features corresponding to DEGs in piglets from both the LR♂ × LW♀ and LW♂ × LR♀ crosses. KEGG pathway information was used in this analysis. The probe set IDs for each category were first mapped to NCBI Entrez gene IDs according to the Agilent porcine array annotation file, and then were mapped to KEGG gene IDs according to the KEGG gene cross-reference file. Pathways that were significantly enriched with DEGs were identified by a hyper-geometric test using R packages (P<0.05, FDR adjusted). Pathways with <3 known porcine genes were discarded. Graphical pathway maps were downloaded from the KEGG FTP server, and DEGs were then highlighted in them according to the coordinate description in the XML files at the KEGG FTP server.

### Quantitative real time RT-PCR analysis

Microarray results for the transcript profiling experiments were validated by real-time quantitative RT-PCR (qRT-PCR) using a SYBR® Premix Ex TaqTM II kit (Takara, Japan). Complementary DNA was synthesized from the same total RNA samples previously used for the microarray analyses. PCR primers were placed at the exon/exon junctions using DNAMAN Software to avoid amplification of any residual genomic DNA, and specificity was determined with BLASTN (Table S1). PCR analyses were performed in triplicate in 20 μl amplification reactions containing 10 μl of 2× SYBR Green PCR Master Mix (Takara, Japan), 0.4 μl ROX II, 20 ng cDNA and 0.4 μl (10mM) of each primer using the following conditions according to the manufacturer's instructions: 94 °C for 5 minutes for 1 cycle, 40 cycles at 94 C for 30 seconds, 58-61 °C for 30 seconds and then 72° C for 30 seconds. Standard curves with 3-fold serial dilutions of cDNA were used to quantify the relative gene expression. Melting curve analysis (60-95°C) was used to assess amplification specificity. GADPH was as the endogenous control gene. Relative expression of each gene was determined using the 2^-ΔΔCt^ method [30]. Statistical analysis was performed to quantify the consistency between the results of the microarray experiments and qRT-PCR.

### Statistical analysis

Body weight, length and height were compared between FD and N group. Meanwhile, muscle cell number, LD fibers diameter and IMTG content were analyzed between not only FD and N group but also LR♂×LW♀ group and LW♂×LR♀group. The above differences in the mean values were compared by the Tukey’s multiple comparison, and means ± S.E.M. are reported. Statistical signiﬁcance was considered as P <0.05. At the same time, the P values for the qRT-PCR data between FD and N group were obtained from Student’s t-test.

## Results

### Body and LD traits of newborn piglets

**Figure 1:**
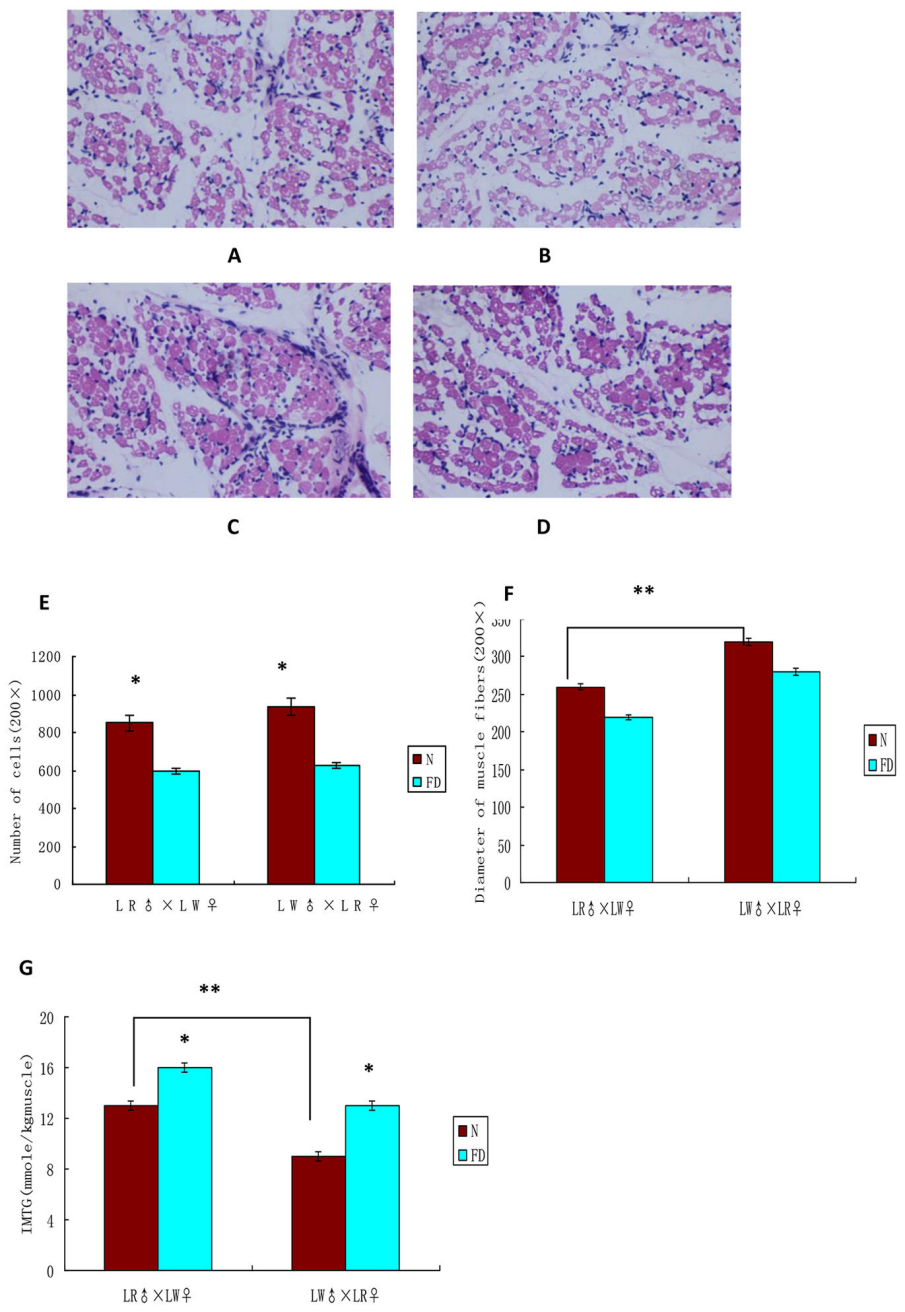
The properties of newborn piglets. A and B indicate HE staining of newborn porcine LD muscle tissue of N and FD group in LR♂×LW♀ cross respectively (200×). C and D indicate HE staining of newborn porcine LD muscle tissue of N and FD group in LW♂×LR♀ cross respectively (200×). E Histogram of muscle cell number in the same visual fields (200×). F Histogram of LD fibers diameter (μm, 200×). G Histogram of intramuscular triglyceride (IMTG) content in LD muscle tissue. Data are means + S.E.M. * denoted significant differences between N and FD groups, and ** denoted significant differences between two N groups in LR♂×LW♀ and LW♂×LR♀ cross (p≤0.05).

The results showed that, in both LR♂×LW♀ and LW♂×LR♀ crosses, birth weight of piglets in the FD group was less than that in N group (P<0.05), while there was no significant difference in body length and body height between FD group and N group (Table 2). It can be seen from HE staining that the LD muscle cross section of newborn piglets contained a lot of skeletal muscle fiber, and blood vessels, nerves and dense connective tissues were around muscle bundle (Figure 1A-D). In the piglets from both the LR♂×LW♀ and LW♂×LR♀ crosses, FD reduced the muscle ﬁber number of piglets (P <0.05, Figure 1A-D, 1E). Fiber diameter of piglets in the LW♂×LR♀ cross was even greater than that of the LR♂×LW♀ cross (Figure 1F), however, it was not different between FD group and N group (Figure 1F). The content of intramuscular triglyceride (IMTG) of LD muscle was significantly higher in piglets from the LR♂×LW♀ cross than the LW♂×LR♀ cross (P <0.05), and was higher in FD group compared with that of N group, especially in the LR♂×LW♀ group (P <0.05) (Figure 1G).

**Table 2:** Growth traits of newborn piglets.

Cross Groups		Litter Size	Body Weight（g)	Body Length（cm)	Body Height（cm)
LR♂×LW♀	N	14	835.4±22.2	22.2±1.4	14.2±3.1
	FD**^*a*^**	12+10**^*b*^**	757.5±35.2*	21.0±3.2	15.3±3.5
LW♂×LR♀	N	13	1069.4±36.3	23.4±2.6	17.2±3.9
	FD**^*a*^**	11+13**^*b*^**	939.6±30.4^*^	22.6±3.8	18.3±3.2

^a^ FD is a folate deficient group and N is a normal folate group.

^b^ The number of birth in FD group is the sum of piglets from two FD sows.

^*^ Compared with N group, difference was statistically significant. p<0.05

### DEGs in the LD muscle of piglets affected by FD during early-mid pregnancy

To analyze DEGs in the LD muscle of piglets that are affected by folate deficiency during early-mid pregnancy, total RNA from LD were isolated, the quality test results of which were shown in Table S2. The RNA quality of 12 samples was suitable and sufficient for use in microarray detection. Microarray analyses focused on two types of genes that were differentially expressed between the FD group and the N group: unique and common. Unique genes are defined as those that are expressed only in the LD muscle of piglets from the LR♂ ×LW♀ cross or the LW♂ × LR♀ cross, but not from both. Common DEGs are defined as those genes that are expressed in the LD muscle of piglets from both the LR♂ × LW♀ and LW♂ × LR♀ crosses, but are expressed at different levels. 

**Figure 2:**
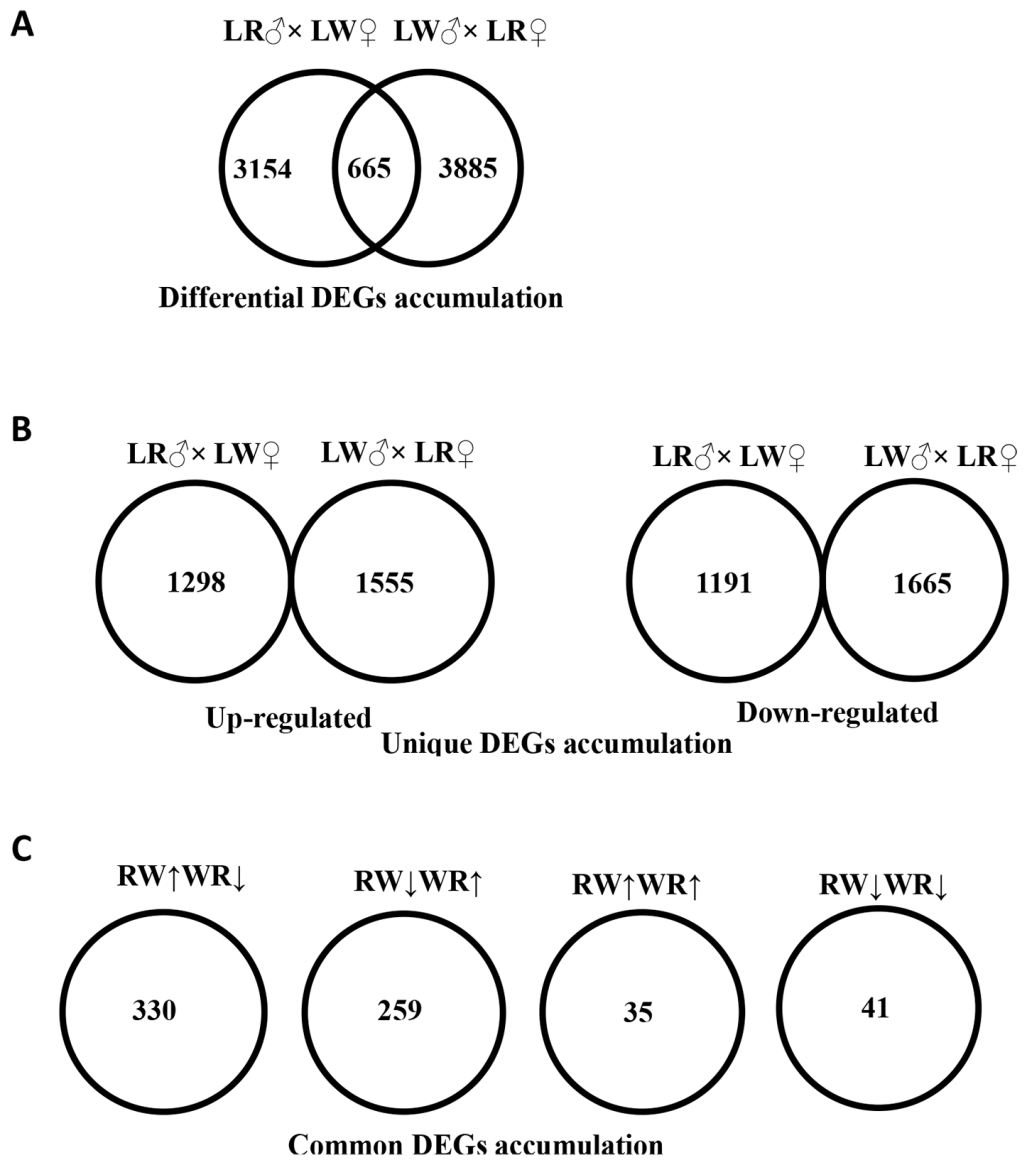
Venn diagrams showing the number of DEGs between the FD and N group by reciprocal cross. A DEGs that displayed differential accumulation in the LR♂ × LW♀ cross and LW♂ × LR♀ cross. B DEGs that display up-regulation and down-regulation uniquely in either LR♂ × LW♀ cross or LW♂ × LR♀ cross. C DEGs that overlap between LR♂ × LW♀ cross and LW♂ × LR♀ cross exhibiting up-regulated (↑) or down-regulated (↓) accumulation (RW is the LR♂ × LW♀ cross and WR is the LW♂ × LR♀ cross).

We detected a total of 3154 transcripts in the LD that were differentially expressed between the FD and the N groups from the LR♂ × LW♀ cross, and 3885 DEGs in the piglets from the LW♂ × LR♀ cross (Figure 2A). The majority of the DEGs were unique genes expressed in LD of piglets from only the LR♂ × LW♀ cross or the LW♂ × LR♀ cross (Figure 2B) , only 665 were common DEGs in the LD muscles of piglets from both crosses (Figure 2A). Among the 665 common DEGs, compared to normal folate group controls, 330 transcripts were highly expressed in the LD muscle of FD piglets from the LR♂ × LW♀ cross while lowly expressed in FD piglets from the LW♂ × LR♀ cross, 259 transcripts were decreased in FD piglets from the LR♂ × LW♀ cross while increased in those from the LW♂ × LR♀ cross, 35 transcripts were increased while 41 transcripts were decreased in FD piglets from both crosses (Figure 2C). 

**Figure 3:**
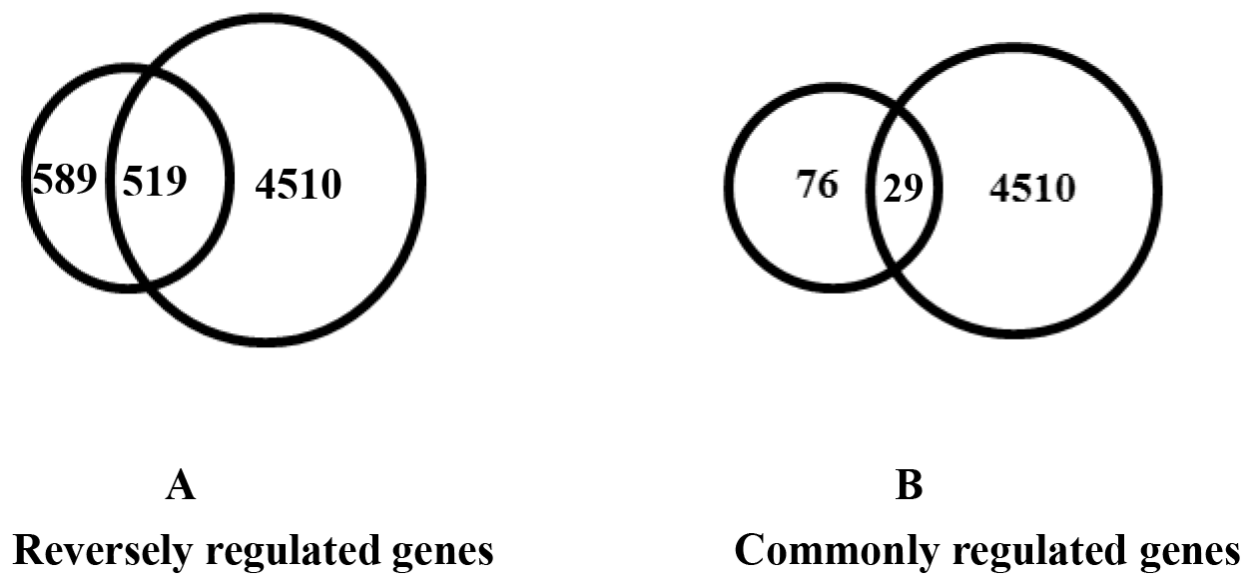
Comparison of the 665 common DEGs with the 4510 DEGs found in the two N groups of the reciprocal cross. **A** Twenty-nine genes out of 76 (35 common up-regulation and 41 common down-regulation) existed in the 4510 genes. **B** 519 out of 589 (665 - 76) exhibited the reverse regulation.

We also observed 4510 DEGs that were differentially expressed in the LD of piglets between LW♂ × LR♀ cross and LR♂ × LW♀ cross of the N group (Figure 3). We compared the 665 common DEGs that are affected by FD with the 4510 DEGs and found that 88.12% (519/589) were overlapped DEGs with expression changes in the reverse direction (Figure 3A), while only 38.16% (29/76) of the ones with expression changes in the same direction (Figure 3B). 

### Known function DEGs affected by folate deficiency during early-mid pregnancy

**Figure 4:**
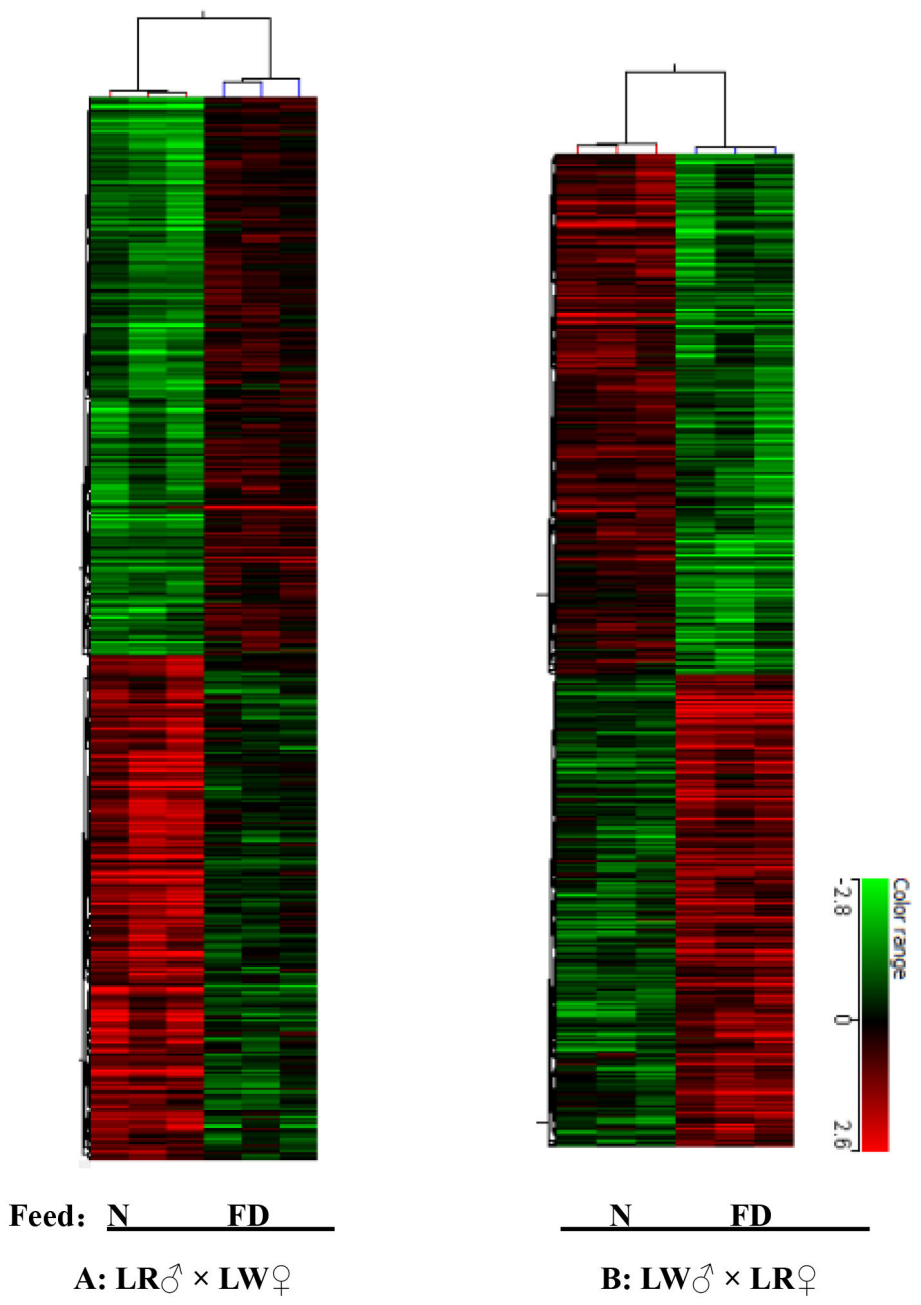
Hierarchical cluster of DEGs between the FD and N group. All the relevant genes are grouped by hierarchical clustering based on expression values across all the samples. Samples are displayed in columns and genes in rows. Red and green represent the increase and decrease of expression, respectively. A Heatmap of DEGs between the folate deficient and normal folate groups within the LR♂ × LW♀ cross. B Heatmap of DEGs within the LW♂ × LR♀ cross. (N: normal diet; FD: folate deficient diet).

Known function genes stand for the most differentially expressed (FC≥2) and informative genes (i.e. with at least one associated GO BP term or a KEGG). In the piglets from the LR♂ × LW♀ cross, 270 known function genes were identified, among which 137 transcripts were up-regulated while 133 transcripts were down-regulated by folate deficiency. In the piglets from the LW♂ × LR♀ cross, 294 known function genes were found, 127 of which were up-regulated while 167 were down-regulated by folate deficiency. Based on the known genes, cluster analysis of all microarrays was performed using the Cluster 3.0 software. The results demonstrated that the expression profiles of three LD samples from the same group piglets were clustered together (Figure 4). The larger the FC, the more the gene expression is affected by folate deficiency. The top ten known function DEGs that are regulated by folate deficiency in piglets from both the LR♂ × LW♀ and LW♂ × LR♀ crosses are listed in Table S3 and Table S4.

### Pathways affected by folate deficiency in the LR♂ × LW♀ and LW♂ × LR♀ crosses

**Figure 5:**
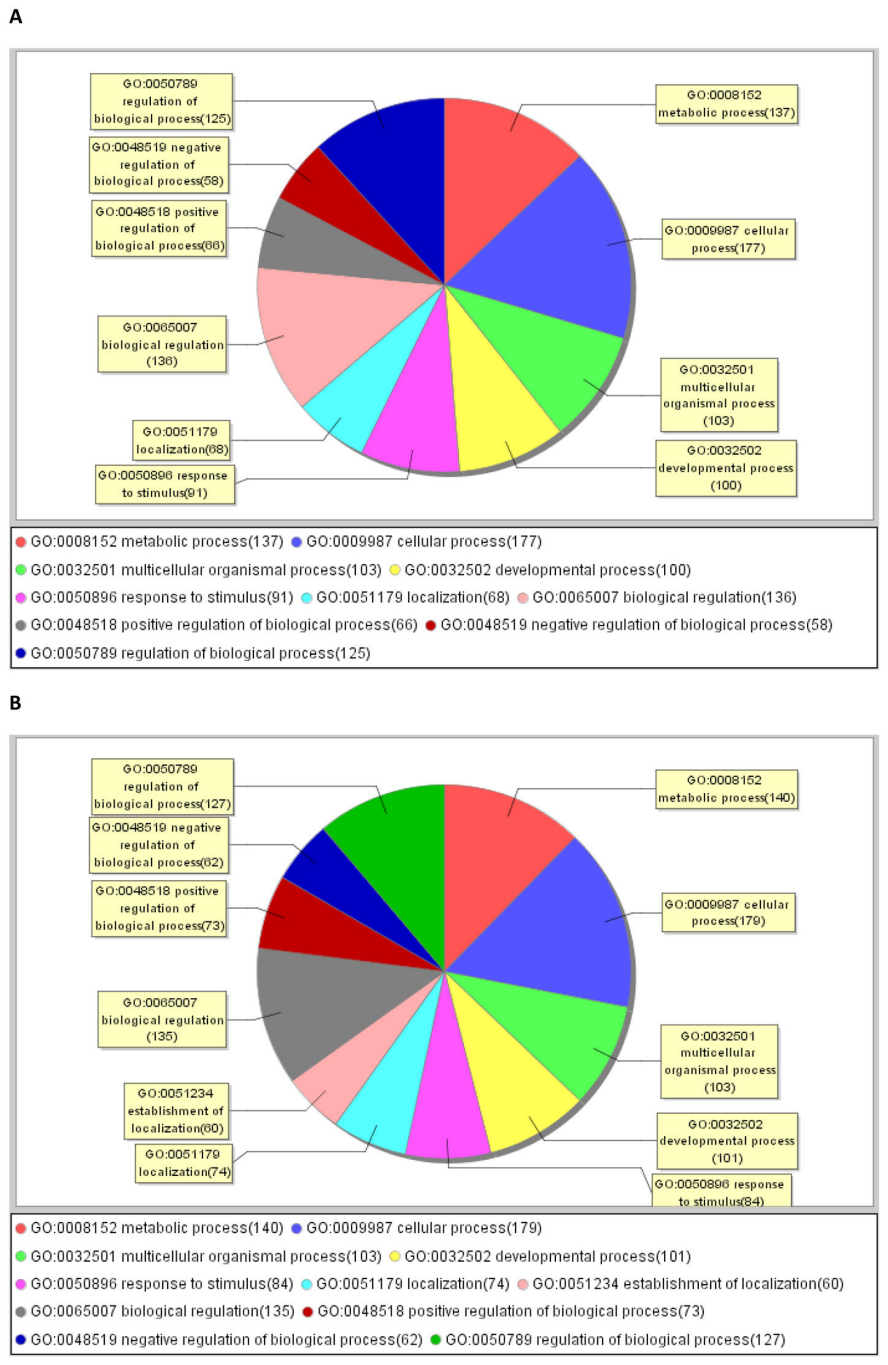
Distribution of DEGs in gene ontology categories. DEGs numbers were in brackets. A LR♂ × LW♀ cross. B LW♂ × LR♀ cross.

To examine whether the DEGs were biologically relevant, further functional pathway analysis was performed using a combination of GO, KEGG and BIOCARTA. In both the LR♂ × LW♀ and LW♂ × LR♀ crosses, the DEGs induced by folate deficiency during early-mid pregnancy affect mainly metabolic and cellular processes, biological regulations, developmental processes, and others (Figure 5A and 5B). The two biological processes that are most differentially affected by FD between the LR♂ × LW♀ and LW♂ × LR♀ cross are multi-organism process (4.27% vs 7.26%) in the up-regulated DEGs (Figure S2) and pigmentation (9.76% vs 2.44%) in the down-regulated DEGs (Figure S3). 

Due to that muscle fiber number (Figure 1E) and IMTG (Figure 1G) of LD were affected by FD, we further analyzed pathways related to skeletal muscle development and fat deposition. Pathways relevant to muscle development that were affected by folate deficiency include the TGF-beta signaling pathway, Toll-like receptor signaling pathway, ErbB signaling pathway, p53 signaling pathway and cell cycle in the piglets from the LR♂ × LW♀ cross (Table 3), Jak-STAT signaling pathway, VEGF signaling pathway and cell adhesion molecules in the piglets from the LW♂ × LR♀ cross (Table 4), and cytokine-cytokine receptor interaction, NOD-like receptor signaling and MAPK signaling pathways in the piglets from both the LR♂ × LW♀ and the LW♂ × LR♀ crosses (Table 3 and 4). Multiple DEGs were involved in lipid metabolism pathways which directly influence fat development and deposition, such as adipocytokine signaling pathway in the piglets from the LR♂ × LW♀ cross (Table 3) and PPAR signaling pathway in the piglets from both the LR♂ × LW♀ and LW♂ × LR♀ crosses (Table 3 and 4). By comparison, only cytokine-cytokine receptor interaction was the common pathway enriched down-regulated genes in piglets from both the LR♂ × LW♀ and LW♂ × LR♀ crosses (Table 3 and 4).

**Table 3:** Pathway annotation of DEGs in piglets from the LR♂ × LW♀ cross.

Affected pathway	Hits(n)	p value**^*b*^**	Genes Involved in Pathway	
	**up-regulation^*a*^**			
Metabolic pathways	13	0.005	AHCY, AMPD, COX6B, DAD1, DLST, DNMT1, HADHA, HYAL1, KMO, ME1, PTGS1, A_72_P179751, A_72_P228947	
Toll-like receptor signaling pathway	4	0.013	IKBKG, LY96, TLR3, TLR7	
PPAR signaling pathway	3	0.027	CD36, CPT1A, FABP4	
Adipocytokine signaling pathway	3	0.020	CD36, CPT1A, IKBKG	
Cytosolic DNA-sensing pathway	3	0.014	CASP1, IKBKG, IL-18	
NOD-like receptor signaling pathway	3	0.011	CASP1, IKBKG, IL-18	
Tryptophan metabolism	3	0.002	CYP1A1, HADHA, KMO	
**down-regulation^*a*^**				
Cytokine-cytokine receptor interaction	12	0	APRIL, BMPR1B, CCL2, CCR1, CCR7, CD40, CXCR2,IL-6,LTA, TNFRSF1A, TNFRSF1B, VEGFA	
Metabolic pathways	11	0.036	ADSSL1, AGPAT6, CTH, CYP2A19, HK2, HYAL2, NOS3, ODC, P4HA1, RDH10, RPA39	
Chemokine signaling pathway	5	0.006	CCL2, CCR1, CCR7, CXCR2, STAT3	
MAPK signaling pathway	4	0.046	GADD45A, MAPK8, MYC, TNFRSF1A	
Focal adhesion	4	0.023	MAPK8, SPP1, THBS1, VEGFA	
Toll-like receptor signaling pathway	4	0.015	CD40, IL6, MAPK8, SPP1,	
Adipocytokine signaling pathway	4	0.003	MAPK8, STAT3, TNFRSF1A, TNFRSF1B	
Cell cycle	3	0.037	A_72_P080381, GADD45A, MYC	
Insulin signaling pathway	3	0.035	HK2, MAPK8, PPP1R3C	
TGF-beta signaling pathway	3	0.020	BMPR1B, MYC, THBS1	
ErbB signaling pathway	3	0.016	HBEGF, MAPK8, MYC	
NOD-like receptor signaling pathway	3	0.012	CCL2, IL6, MAPK8	
p53 signaling pathway	3	0.012	GADD45A, PMAIP1, SERPINE1	
Arginine and proline metabolism	3	0.008	NOS3, ODC, P4HA1	

***^a^*** “Up-regulation” and “down-regulation” indicate differentially expressed genes between the FD group and N groups in the LR♂ × LW♀ cross. ***^b^*** P value, enrichment test P value ≤0.05.

**Table 4:** Pathway annotation of DEGs in piglets from the LW♂ × LR♀ cross.

Affected pathway	Hits(n)		p value	Genes Involved in Pathway
**up-regulation^*a*^**				
Cytokine-cytokine receptor interaction		8	4E-04	AMCF-II, CXCL2, GH1, IL6, LEP, LTA, TNFRSF8, VEGFA
MAPK signaling pathway		7	3E-04	ATF4, BDNF, CASP3, Hsp27, HSP70, HSP70.2
Endocytosis		4	0.025	HSP70, HSP70.2, SLA-3, SLA-7
Jak-STAT signaling pathway		4	0.018	GH1, IL6, LEP, STAT3
Focal adhesion		4	0.018	THBS1, VCL, VEGFA, VTN
Cell adhesion molecules (CAMs)		4	0.009	ICAM-1, SELE, SLA-3, SLA-7
Spliceosome		3	0.019	HSP70, HSP70.2,
ErbB signaling pathway		3	0.013	AREG, HBEGF, TGFA
NOD-like receptor signaling pathway		3	0.009	HSP90AA1, HSP90B1, IL6
Arginine and proline metabolism		3	0.006	ARG1, P4HA1, A_72_P090121
VEGF signaling pathway		3	0.006	Hsp27, NFATC1, PGHS-2
**down-regulation^*a*^**				
Cytokine-cytokine receptor interaction		6	0.028	A_72_P223827, A_72_P288509, A_72_P198177, CXCL12, IL15, TNFSF10
PPAR signaling pathway		5	0.002	ANGPTL4, APOA1, CPT1B, DBI, FABP7
Glutathione metabolism		3	0.012	A_72_P284744, ANPEP, MGST3
Metabolism of xenobiotics by cytochrome P450		3	0.009	A_72_P284744, CYP1A1, MGST3

^a^ “Up-regulation” and “down-regulation” indicate differentially expressed genes between the folate deficient and normal folate groups in the LW♂ × LR♀ cross.

### Different genes are affected in piglets from the LR♂ × LW♀ and LW♂ × LR♀ crosses

According to GO and KEGG pathway analysis, candidate genes regulated by folate deficiency during early-mid pregnancy were screened using the keywords cell development and fatty acid metabolism. Full details of some candidate genes, probgene ID, GeneID, description, identification, and FC Absolute were listed in Table 5. The growth and development related genes VEGFA, STAT3, IFRD1, and IL-6 are down-regulated in the piglets from the LR♂ × LW♀ cross but up-regulated in the ones from LW♂ × LR♀ cross; FST, PTGS1, IL-15 are just the reverse. Most of the fat metabolism-related genes regulated by folate deficiency are different in the piglets from the LR♂ × LW♀ and LW♂ × LR♀ crosses. For instance, MAPK8, ME1, FABP4, and C/EBPα are regulated in the piglets from LR♂ × LW♀ cross but unchanged in the ones from the LW♂ × LR♀ cross; whereas CYP1A1, CPT1B, LDLR are regulated in the piglets from LW♂ × LR♀ group but are unchanged in the ones from the LR♂ × LW♀ cross (Table 5).

**Table 5:** Candidate genes regulated by FD during early-mid pregnancy.

ProbeId	GeneId	Gene Symbol	LR♂ × LW♀FC**^*a*^**	LW♂ × LR♀FC**^*a*^**	Gene Description
A_72_P178121	1E+08	ADSSL1	-2.7297**^*c*^**	-3.8056	adenylosuccinate synthase like 1
A_72_P110291	492279	HSPD1	+2.3501**^*c*^**	+4.1358	heat shock 60kDa protein 1
A_72_P387383	396708	lc8	+2.1121	+3.2866	cytoplasmic light-chain dynein
A_72_P116846	397157	VEGFA	-2.2821	+2.1492	vascular endothelial growth factor A
A_72_P349223	733648	STAT3	-4.4453	+2.5742	signal transducer and activator of transcription 3
A_72_P035786	493185	IFRD1	-9.2344	+3.3435	interferon-related developmental regulator 1
A_72_P177826	399500	IL-6	-30.8833	+7.2649	interleukin 6
A_72_P088416	445002	FST	+3.1351	-4.6879	follistatin
A_72_P360403	397541	PTGS1	+2.7250	-3.2205	prostaglandin-endoperoxide synthase 1
A_72_P035856	397683	IL-15	+3.1045	-2.134	interleukin 15
A_72_P290469	396610	MAPK8	-2.1780	NS**^*b*^**	mitogen-activated protein kinase 8
A_72_P306288	397538	ME1	+3.4203	NS	malic enzyme 1
A_72_P127301	399533	FABP4	-3.5135	NS	fatty acid binding protein 4, adipocyte
A_72_P174066	606746	DNMT1	+2.0254	NS	DNA -methyltransferase 1
A_72_P344038	397307	C/EBPa	-2.5524	NS	CCAAT/enhancer binding protein (C/EBP), alpha
A_72_P349423	397015	PTTG1	NS	-3.3327	pituitary tumor-transforming 1
A_72_P223362	403103	CYP1A1	NS	-3.2321	carnitine palmitoyl transferase 1A
A_72_P232387	399528	CPT1B	NS	-2.1914	carnitine palmitoyl transferase 1B
A_72_P306144	397484	TGFA	NS	+2.0746	transforming growth factor, alpha
A_72_P255277	396801	LDLR	NS	+2.3278	low density lipoprotein receptor

^a^ FC is the abbreviation of fold change value, FC is the expression ratio of differentially expressed genes between folate deficiency group and normal diet group.

^b^ NS: nonsense, FC<2

^c^ “-”and “+” indicate down-regulated and up-regulated by folate deficiency, respectively

### Validation of microarray analysis by quantitative RT-PCR

**Figure 6:**
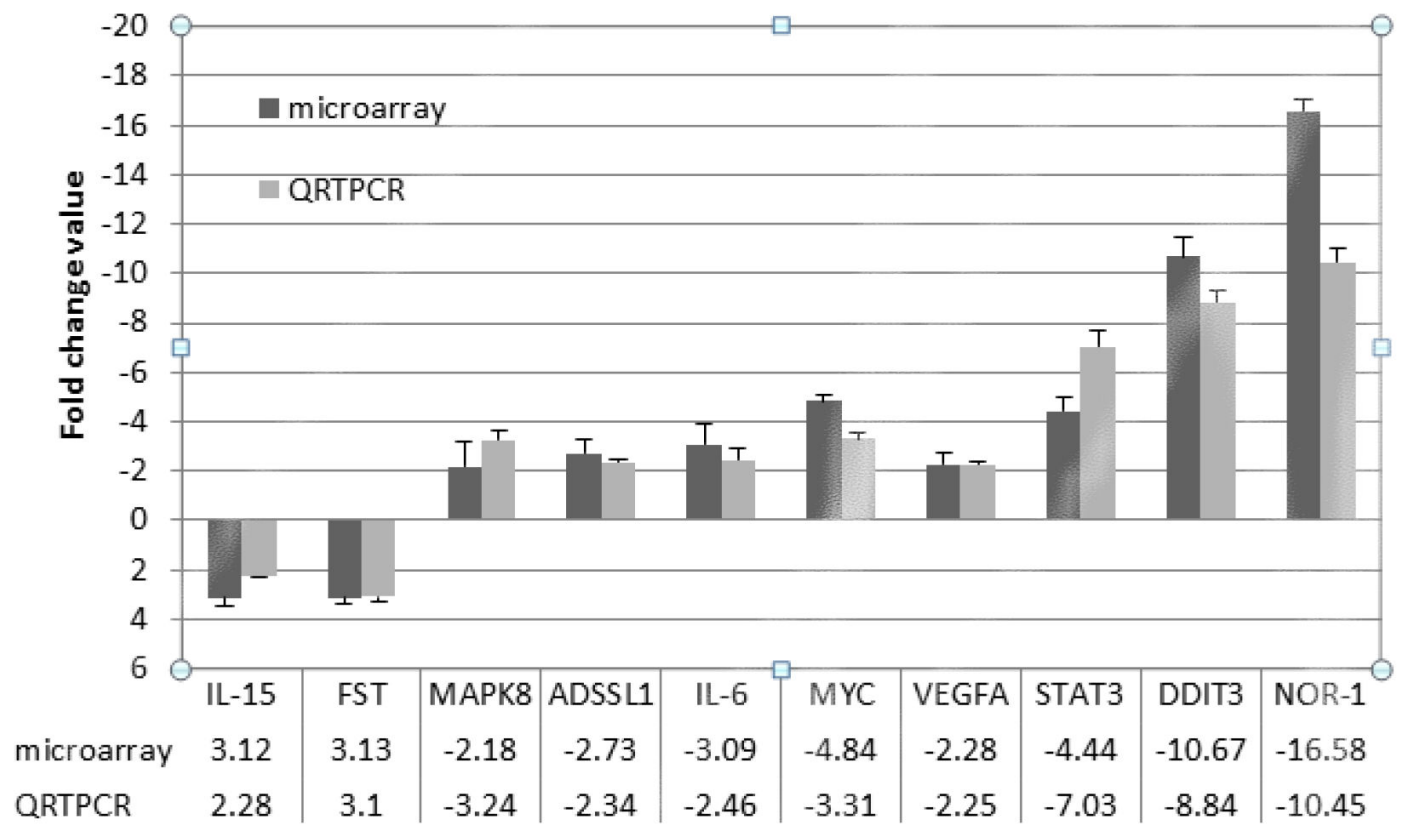
Validation of microarray data by quantitative RT-PCR. The fold change value is expressed as the expression ratio of the FD group to N group during early-mid pregnancy in the LR♂ × LW♀ cross. Statistical significances are reported below the plot as P values for the microarray data and as Student’s t-test P values for the qRT-PCR data.

Ten genes (ADSSL1, NOR-1, VEGFA, MAPK8, STAT3, MYC, FST, DDIT3, IL-15, IL-6) were selected to validate the microarray data by real-time quantitative RT-PCR (qRT-PCR). Collectively, the results indicated that the expression patterns of these genes were consistent within the microarray data. Although the magnitude of expression is somewhat different between microarray and qRT-PCR, the direction of the regulation of expression was the same between the two techniques (Figure 6).

## Discussion

In this study, the skeletal muscle transcriptome of piglets affected by folate deficiency were investigated in a reciprocal cross between Landrace and Laiwu pigs. During development, the muscle ﬁber number has been determined in the embryonic period and no longer increases after birth, so the fetal period is crucial for skeletal muscle development [31]. Porcine myogenesis is divided into the primary and secondary processes, which takes place from d 30 to d 60, and from d 54 to d 90 of gestation, respectively [32], together determines the muscle fiber number in individual development [33]. We found that, during pregnancy from start to 60 days, muscle fiber number of piglets was reduced by folate deficiency from both the LR♂×LW♀ and LW♂×LR♀ crosses. Two previous studies also reported that sow under-nutrition decreased muscle fiber number in the porcine fetus and on subsequent postnatal growth [34,35]. Similarly, Zhu et al. found that nutrient restriction in pregnant ewes from early to mid-gestation reduced the number of fetal skeletal muscle ﬁbers [36]. Meanwhile, we found that the birth weight of newborn piglets in FD group was less than in N group. Studies in human have shown that folate deficiency during pregnancy can cause fetal IUGR and lower birth weight [20]. Skeletal muscle normally represents 35-40% of the body weight in newborns, and IUGR is always associated with development dysplasia of muscle tissue [37]. Reduced myoﬁber number in IUGR newborns limits the ability for postnatal compensatory development of skeletal muscle [21,37]. The results of this study suggested that folate deficiency during early-mid pregnancy likely affected individual growth of adult pigs by limiting the growth and development of fetal muscle tissue. 

Myogenesis is a complex well-organized prenatal process involving proliferation and differentiation of myoblasts that are regulated by complex gene network [38]. In this study, using microarray technology, we investigated the difference in the piglet muscle-speciﬁc transcriptome proﬁles of FD group and N group to uncover genes underlying skeletal muscle development that are affected by folate deficiency. The results showed that a large number of DEGs existed between the FD and N groups, including genes involved in many biological processes, such as metabolic, cellular, and developmental processes, growth, biological adhesion both in the LR♂ × LW♀ cross and in the LW♂ × LR♀ cross, implying that folate deficiency during early-mid pregnancy indeed impacted muscle gene transcription and expression. 

This study also revealed that the content of intramuscular triglyceride was affected by FD, and furthermore, the effect was different between piglets from the LR♂ × LW♀ and LW♂ × LR♀ crosses. Consistent with this, microarray data showed that lipid metabolism-related genes and associated metabolic pathways in piglets were significantly affected by folate deficiency of the pregnant sows. Kumar et al. testified that maternal dietary folate restrictions can alter adiposity and lipid metabolism in Wistar rat offspring [39]. Notably, we also found that folate deficiency regulated lipid metabolism via different signaling pathways between the piglets from the LR♂ × LW♀ and LW♂ × LR♀ crosses and more extensively in piglets from the LR♂ × LW♀ cross than in piglets from the LW♂ × LR♀ cross. In piglets from the LW♂ × LR♀ cross, ANGPTL4, APOA1, CPT1B, DBI, and FABP7 genes were down-regulated by FD via the PPAR signaling pathway, while in piglets from the LR♂ × LW♀ cross, more genes were regulated, for example, CD36, CPT1A, FABP4, IKBKG via the PPAR signaling pathway and adipocytokine signaling pathway are up-regulated; MAPK8, STAT3, TNFRSF1A, TNFRSF1B via adipocytokine signaling pathway are down-regulated (Table 3 and Table 4). 

That different DEGs, pathways and genes were affected by FD in piglets from the LR♂ × LW♀ and LW♂ × LR♀ crosses as mentioned above is intriguing. We also compared the LD transcriptome of piglets between the LR♂ × LW♀ and LW♂ × LR♀ crosses of N group and found that 4510 DEGs exist between piglets from the two crosses. We speculate that two factors may cause such differences. One factor is that different parental pigs were used in the reciprocal cross. In the LR♂ × LW♀ and LW♂ × LR♀ crosses, LW and LR sows are used, respectively. The LW pigs have an extremely high intramuscular fat content as compared to the LR pigs. Coincidentally, the content of intramuscular triglyceride of LD muscle of piglets from the LR♂ × LW♀ cross was significantly higher than from the LW♂ × LR♀ cross and are significantly affected by FD. Similar studies demonstrated that fat deposition has maternal inheritance [40,41], that the phenotype of the individual is not only related to their environment and genotype, but also to their mothers’ environment and phenotype [42]. 

Due to that folate is an essential nutrient required for S-adenosyl methionine synthesis which is a methyl donor for DNA methylation and histone methylation modifications, the difference is also likely caused by epigenetic factors. In this study, to test whether transcriptome changes affected by FD are caused by epigenetic factors, in the experimental design, we used a reciprocal cross between full-sibling LW and full-sibling LR pigs to minimize genetic differences. To find the genes most likely controlled by epigenetic factors, we first compared DEGs affected by FD in either LR♂ × LW♀ cross or the LW♂ × LR♀ cross, then compared the commonly affected genes with the 4510 DEGs between the two crosses of N group. By this way, we found that folate deficiency affected about 3000 different unique genes in each cross and more than 500 common genes between the two crosses (Figure 3). The fact that different pathways and genes are affected by FD in the LD of piglets between the LR♂ × LW♀ and LW♂ × LR♀ crosses implies that epigenetic mechanisms are involved in myogenesis and intramuscular fat deposition of porcine fetus, of which imprinted genes, the expression of which depends on whether paternally or maternally inherited allele, are most likely affected by FD via changing the methylation status of their DNA and/or histones. Several studies reported the existence of imprinted genes underlying myogenesis and fat deposition, for example, Nezer et al. identified an imprinted quantitative trait loci (QTL) with major effect on muscle mass and fat deposition that maps to the IGF2 locus in pigs [43], a genome-wide, significant, paternally expressed QTL is located on SSC2 with the best position at 63 cM [44] and chromosome 6 harbored a maternally expressed QTL on the short arm and a paternally expressed QTL on the long arm, both affecting intramuscular fat content [45]. Our data on LD transcriptome analysis affected by FD in the reciprocal pig crosses provide further evidence of genomic imprinting that controls myogenesis and fat deposition.

In the process of embryonic development, folate has a unique function as a methyl donor for nucleotide synthesis, amino acid synthesis, and biological methylation in the form of a coenzyme [46-48]. From our transcriptome results, it can be seen that, these effects are widespread and almost all biological processes were affected in the newborn body. McKay demonstrated that early life folate depletion affected epigenetic marks, and that altered epigenetic marks persisted into adulthood and were not modulated by post-weaning folate supply [49]. The epigenetic effect of FD on genes underlying myogenesis and intramuscular fat deposition in piglets requires further study. 

## Conclusion

Our genome-wide microarray results show that folate deficiency in sows during early-mid pregnancy alters the transcriptome of the longissimus dorsi muscle in the offsprings, thus affecting the process of myogenesis and IMF deposition in fetal pigs. From functional analyses, sow folate deficiency affected almost all biological processes in the progeny. Three affected molecular pathways, including metabolic pathways, muscle development pathways and lipid metabolism pathways, were identified. Lipid metabolism-related genes and associated metabolic pathways in piglets were extensively regulated by folate deficiency of the pregnant sow, especially in the LR♂ × LW♀ cross. This study provides evidence that sow folate nutrition affects skeletal muscle transcriptome of the offspring, which is likely caused by altering epigenetic modifications.

## Supporting Information

Figure S1 **The license of the experimental animal.**
(TIF)

Figure S2 **Functional categorization of the probe sets that displayed differential accumulation (percent of hits) up-regulated by folate deficiency during early-mid pregnancy in the LR♂ × LW♀ and LW♂ × LR♀ crosses.**
(TIF)

Figure S3 **Functional categorization of the probe sets that displayed differential accumulation (percent of hits) down-regulated by folate deficiency during early-mid pregnancy in the LR♂ × LW♀ and LW♂ × LR♀ crosses.**
(TIF)

Table S1 **PCR primers of qRT-PCR.**
(DOCX)

Table S2 **Total RNA 2100 QC report of 12 samples.**
(DOCX)

Table S3 **Top ten DEGs according to FC value in the LR♂ × LW♀ cross.**
(DOCX)

Table S4 **Top ten DEGs according to FC value in the LW♂ × LR♀ cross.**
(DOCX)
