# Supplementary figures and images for: Folate Deficiency during Early-Mid Pregnancy Affects the Skeletal Muscle Transcriptome of Piglets from a Reciprocal Cross
Source: PLoS One. 2013 Dec 9;8(12):e82616. doi: 10.1371/journal.pone.0082616 (PMC3857258; doi:10.1371/journal.pone.0082616)

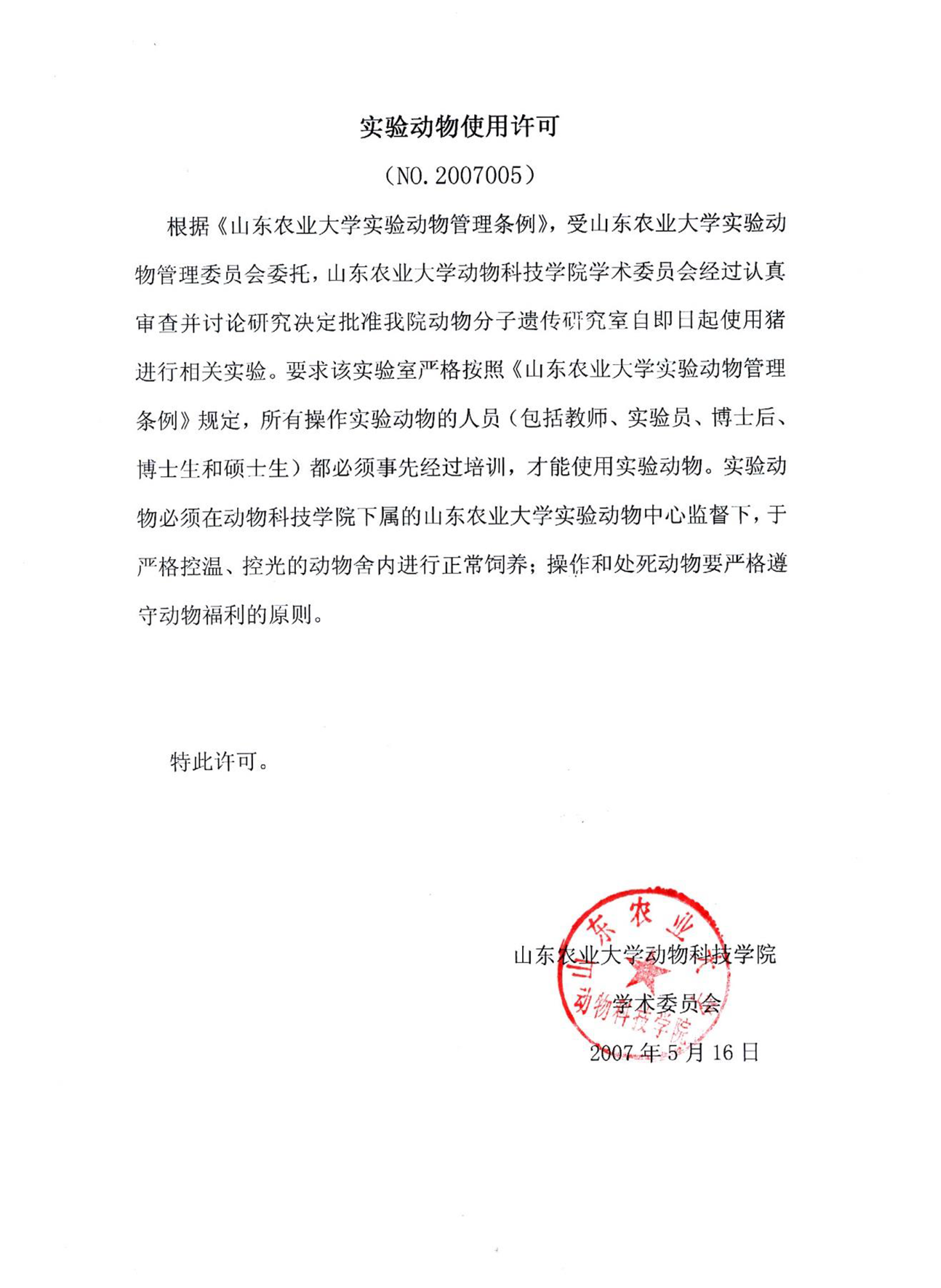

Supplement: Figure S1 — The license of the experimental animal. (TIF) [file pone.0082616.s001.tif]

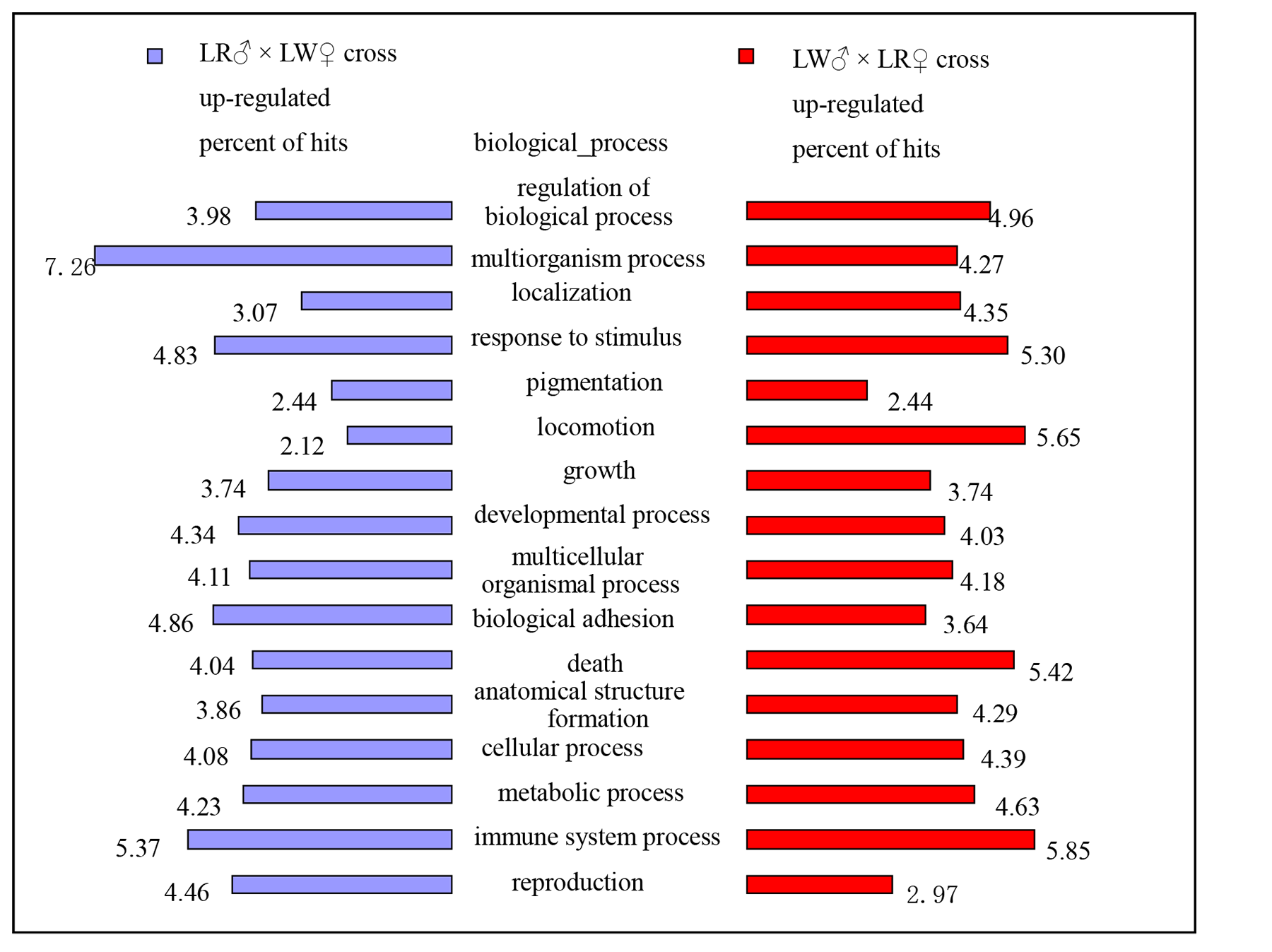

Supplement: Figure S2 — Functional categorization of the probe sets that displayed differential accumulation (percent of hits) up-regulated by folate deficiency during early-mid pregnancy in the LR♂ × LW♀ and LW♂ × LR♀ crosses. (TIF) [file pone.0082616.s002.tif]

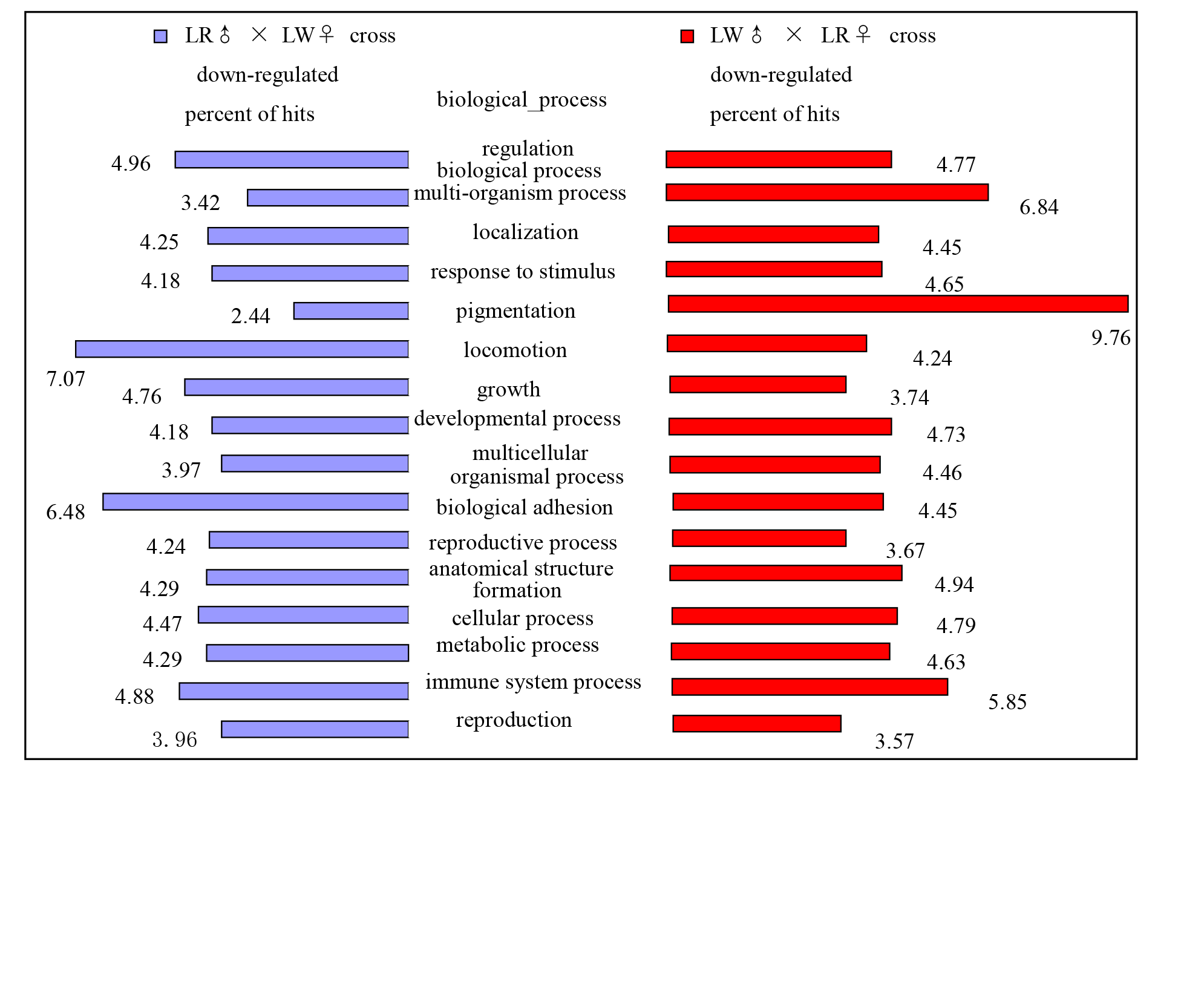

Supplement: Figure S3 — Functional categorization of the probe sets that displayed differential accumulation (percent of hits) down-regulated by folate deficiency during early-mid pregnancy in the LR♂ × LW♀ and LW♂ × LR♀ crosses. (TIF) [file pone.0082616.s003.tif]
