# Supplementary material for: Folate Deficiency during Early-Mid Pregnancy Affects the Skeletal Muscle Transcriptome of Piglets from a Reciprocal Cross
Source: PLoS One. 2013 Dec 9;8(12):e82616. doi: 10.1371/journal.pone.0082616 (PMC3857258; doi:10.1371/journal.pone.0082616)
Supplement: Table S1 — PCR primers of qRT-PCR. (DOCX) [file pone.0082616.s004.docx]

**Table S1. PCR primers of qRT-PCR**

| Gene | Primer5’-3’ | Amplicon  size(bp) | Annealing temprature(℃) |
| --- | --- | --- | --- |
| ADSSL1-F | 5'AGGTCTATGGCGTGGTGAAG3' |  |  |
| ADSSL1-R | 5'ATGTGGGCGTATCTCAGGA3' | 178 | 60 |
| NOR-1-F | 5'CAGAAGTGTCTCAGTGTCGG3' |  |  |
| NOR-1-R | 5'AACGGGCTCTTTGGTTTG3' | 97 | 60 |
| VEGF-F | 5'GGAGTTCAACATCGCCAT3' |  |  |
| VEGF-R | 5'CTTGCCTCGCTCTATCTTTC3' | 125 | 58 |
| MAPK8-F | 5'TGGATGAAAGGGAACACAC3' |  |  |
| MAPK8-R | 5'CAGACGATGATGACGATGG3' | 162 | 59 |
| STAT3-F | 5'GCAAGACCTGAATGGAAACA3' |  |  |
| STAT3-R | 5'CCAACTCACTCACGATGCTT3' | 110 | 61 |
| MYC-F | 5'GGACGCTGGATTTCCTTCG3' |  |  |
| MYC-R | 5'TGCTGCTGCTGCTGGTAGA3' | 154 | 60 |
| FST-F | 5'TACTGTTCTGAGGAGGTGG3' |  |  |
| FST-R | 5'AGATGTAAAGAGCAGCCG3' | 177 | 60 |
| DDIT3-F | 5'TGAGTCATTGCCTTTCTCC3' |  |  |
| DDIT3-R | 5'AGGGTCAAGAGTGGTGAAG3' | 160 | 58 |
| IL-15-F | 5'TCCAGTGCTACTTGTGTTTAC3' |  |  |
| IL-15-R  IL-6-F  IL-6-R | 5'ACTTCATCGCTGTTACTTTG3'  5'CCACCAGGAACGAAAGAGA3'  5'CAGTAGCCATCACCAGAAGC3' | 230  120 | 59  60 |
| GADPH-F | 5'ACTCACTCTTCCACTTTTGATGC3' |  |  |
| GADPH-R | 5'TGTTGCTGTAGCCAAATTCA3' | 100 | 61 |

“F” is upstream primer sequence, “R” is downstream primer sequence.

GADPH is housekeeping gene as a control.
