# Supplementary material for: Folate Deficiency during Early-Mid Pregnancy Affects the Skeletal Muscle Transcriptome of Piglets from a Reciprocal Cross
Source: PLoS One. 2013 Dec 9;8(12):e82616. doi: 10.1371/journal.pone.0082616 (PMC3857258; doi:10.1371/journal.pone.0082616)
Supplement: Table S2 — Total RNA 2100 QC report of 12 samples. (DOCX) [file pone.0082616.s005.docx]

**Table S2. Total RNA 2100 QC Report of 12 Samples**

| Sample Name | Vol.  (μL) | Con. (μg/μL) | Amount  (μg) | A260/ A280 | 2100 RIN | | 28S/18S | |
| --- | --- | --- | --- | --- | --- | --- | --- | --- |
| 1 | 200 | 0.957 | 191.4 | 1.74 | 8.2 | 1.8 | |  |
| 2 | 200 | 0.668 | 133.6 | 1.76 | 8.2 | 1.8 | |  |
| 3 | 200 | 0.864 | 172.9 | 1.73 | 8.3 | 2.1 | |  |
| 4 | 200 | 0.911 | 182.2 | 1.85 | 8.6 | 1.8 | |  |
| 5 | 200 | 0.965 | 193.0 | 1.83 | 8.6 | 1.9 | |  |
| 6 | 200 | 0.549 | 109.8 | 1.85 | 8.6 | 2.0 | |  |
| 7 | 200 | 0.394 | 78.7 | 1.79 | 8.2 | 2.0 | |  |
| 8 | 200 | 1.084 | 216.9 | 1.78 | 8.5 | 1.9 | |  |
| 9 | 200 | 0.953 | 190.5 | 1.79 | 8.4 | 2.1 | |  |
| 10 | 200 | 0.530 | 105.9 | 1.91 | 8.2 | 1.9 | |  |
| 11 | 200 | 0.562 | 112.3 | 1.87 | 8.5 | 2.0 | |  |
| 12 | 200 | 0.657 | 131.3 | 1.90 | 8.6 | 2.1 | |  |
