# Supplementary material for: Folate Deficiency during Early-Mid Pregnancy Affects the Skeletal Muscle Transcriptome of Piglets from a Reciprocal Cross
Source: PLoS One. 2013 Dec 9;8(12):e82616. doi: 10.1371/journal.pone.0082616 (PMC3857258; doi:10.1371/journal.pone.0082616)
Supplement: Table S3 — Top ten DEGs according to FC value in the LR♂ × LW♀ cross. (DOCX) [file pone.0082616.s006.docx]

**Table S3. Top ten DEGs according to FC value in the LR♂ × LW♀ cross**

| Gene Symbol | | FC^a^ | | P-value | Description | RefSeq |
| --- | --- | --- | --- | --- | --- | --- |
| **Up-regulated** | |  | |  |  |  |
| LOC733603 | | 26.6387 | | 0.0377 | serum amyloid A2 | NM_001044552 |
| UCHL1 | | 11.9779 | | 8.91E-05 | Ubiquitin carboxyl-terminal esterase L1 | NM_213763 |
| LOC100152391 | | 10.6757 | | 0.0043 | similar to glutamate receptor, ionotrophic | XM_001924371 |
| C3 | | 10.4160 | | 0.0323 | complement component 3 (C3) | NM_214009 |
| LOC100152966 | | 9.4578 | | 0.0129 | similar to lipoma HMGIC fusion-partner-like 1 | XM_001924418 |
| CD180 | | 7.7067 | | 0.0028 | CD180 molecule | NM_214357 |
| IRG6 | | 6.9756 | | 0.0471 | inflammatory response protein 6 | NM_213817 |
| LOC100156239 | | 6.6676 | | 9.67E-04 | similar to immunoglobulin domain 4 precursor | XM_001924625 |
| LOC100153917 | | 6.1030 | | 0.0425 | similar to slit and trk like 6 | XM_001924423 |
| PSMD4 | | 5.9511 | | 0.0105 | proteasome 26S subunit, non-ATPase, 4 | NM_001044535 |
| **Down-regulated** |  | |  | | |  |
| SERPINE1 | | 91.5306 | | 0.0022 | serpin peptidase inhibitor clade | NM_213910 |
| HK2 | | 32.5107 | | 4.51E-04 | hexokinase 2 | NM_001122987 |
| IL6 | | 30.8833 | | 0.0310 | interleukin 6 | NM_214399 |
| NOR-1 | | 22.0353 | | 0.0364 | neuron-derived orphan receptor-1 alfa | NM_214247 |
| DDIT3 | | 20.6710 | | 0.0044 | DNA-damage-inducible transcript 3 | CK_454369 |
| NPG4 | | 20.4672 | | 0.0347 | protegrin 4 | NM_213863 |
| PMAP-23 | | 19.9113 | | 0.0377 | rnbm14b_e11.y1 nbm Sus scrofa cDNA |  |
| SOX9 | | 19.4472 | | 0.0339 | SRY (sex determining region Y)-box 9 | NM_213843 |
| CCL2 | | 19.3457 | | 0.0074 | chemokine (C-C motif) ligand 2 | NM_214214 |
| STC2 | | 17.4804 | | 0.0257 | stanniocalcin 2 | NM_001110173 |

^a^ Fold change value is expressed as the expression ratio of folate deficiency group and normal diet group samples.
