# Supplementary material for: Folate Deficiency during Early-Mid Pregnancy Affects the Skeletal Muscle Transcriptome of Piglets from a Reciprocal Cross
Source: PLoS One. 2013 Dec 9;8(12):e82616. doi: 10.1371/journal.pone.0082616 (PMC3857258; doi:10.1371/journal.pone.0082616)
Supplement: Table S4 — Top ten DEGs according to FC value in the LW♂ × LR♀ cross. (DOCX) [file pone.0082616.s007.docx]

**Table S4. Top ten DEGs according to FC value in the LW♂ × LR♀ group**

| Gene Symbol | FC^a^ | P-value | Description | | RefSeq |
| --- | --- | --- | --- | --- | --- |
| **up-regulated** |  |  |  |  |  |
| HSP70 | 433.9440 | 6.73E-07 | heat shock protein 70 | | NM_001123127 |
| NOR-1 | 243.9533 | 0.006236 | neuron-derived orphan receptor-1 alfa | | NM_214247 |
| HSP70.2 | 188.5077 | 9.39E-04 | heat shock protein 70.2 | | NM_213766 |
| HSPH1 | 147.6109 | 4.61E-05 | heat shock 105kDa/110kDa protein 1 | | NM_001097504 |
| DNAJB1 | 48.0197 | 1.58E-05 | OVRM10011B04, expressed in ovary | |  |
| LAG3 | 36.6203 | 0.0070 | lymphocyte-activation gene 3 | | NM_001105306 |
| GH1 | 26.9784 | 0.0371 | growth hormone (GH1) | | NM_213869 |
| CHI3L1 | 26.4971 | 0.0039 | chitinase 3-like 1(CHI3L1) | | NM_001001540 |
| GP38K | 26.4972 | 0.0040 | 38 kDa heparin-binding glycoprotein | |  |
| SELE | 26.1134 | 0.0064 | selectin E | | NM_214268 |
| **down-regulated** |  |  |  | |  |
| MYH4 | 52.7792 | 0.0153 | myosin, heavy chain 4, skeletal muscle | | NM_001123141 |
| IGG2B | 17.2499 | 0.0218 | clone:SPL010013G04, expressed in spleen | | NM_237225 |
| TOPOII | 16.2724 | 0.0140 | topoisomersae II (TOPOII) | | NM_213884 |
| CCNB1 | 11.8032 | 0.0165 | cyclin B (CCNB1) | | NM_001170768 |
| LOC100153917 | 10.2817 | 0.0063 | similar to slit and trk like 6 | | XM_001924423 |
| TNFSF10 | 9.4496 | 0.0153 | tumor necrosis factor superfamily, member 10 | | NM_001024696 |
| KCNS3 | 9.1568 | 0.0272 | potassium voltage-gated channel, delayed-rectifier, subfamily S | | NM_001044596 |
| LOC396781 | 9.1510 | 0.0306 | IgG heavy chain | | NM_213828 |
| CDK1 | 8.3583 | 0.0010 | cyclin-dependent kinase 1 | | NM_001159304 |
| RHCG | 8.3500 | 0.0208 | Rh family, C glycoprotein | | NM_001044577 |

^a^ FC is the abbreviation of fold change value, FC is the expression ratio of differentially expressed genes between folate deficiency group and normal diet group.
